# Complete genome sequence of *Bacillus thuringiensis* strain RC340, isolated from a temperate forest soil sample in New England

**DOI:** 10.1128/MRA.00607-23

**Published:** 2023-10-31

**Authors:** Brendan Sullivan, Claire E. Kitzmiller, Wyatt C. Tran, Mallory Choudoir, Rachel Simoes, Nipuni Dayarathne, Kristen M. DeAngelis

**Affiliations:** 1 Department of Microbiology, University of Massachusetts, Amherst, Massachusetts, USA; 2 Department of Plant and Microbial Biology, North Carolina State University, Raleigh, North Carolina, USA; University of Maryland School of Medicine, Baltimore, Maryland, USA

**Keywords:** environmental microbiology, DNA sequencing, climate change, soil microbiology, microbiology, genome analysis

## Abstract

The complete genome sequence of *Bacillus thuringiensis* strain RC340, isolated from an environmental microbiology experiment soil sample is presented here. *B. thuringiensis* strain RC340 sequenced by GridION consists of a single genome consisting of 5.86 million bases, 8,152 predicted genes, and 0.23% contamination.

## ANNOUNCEMENT

Soil microenvironments are highly competitive, and bacteria challenge one another for the limited resources available. *B. thuringiensis* has several mechanisms to gain a competitive advantage including bacteriocin production and antimicrobial resistance (AMR) proteins (1). Soil warming may induce the prevalence of AMR genes (2), so the focus of our genomics analysis was gene signatures of bacitracin resistance mechanisms.

Soils from the Harvard Forest soil warming experiment were experimentally heated 5°C above ambient temperature with unheated controls (3). Soil containing RC340 was isolated in 2022 from a heated soil plot, and then a streak of the soil sample was grown on actinobacteria isolation agar (AIA) (4) with 100 mg of L-1 cycloheximide. The isolate colony was then selected and grown on 10% tryptic soy broth at 30°C with shaking at 150 rpm until cultures reached an OD of 0.5, then spun at 4,000 rpm for 15 min. Genomic DNA was extracted from cell pellets using the CTAB method (5). 16S rRNA sequence was sequenced as previously described (6) to determine the genome size of the species with the highest percent identity and estimate the target 40X coverage for the genome assembly. DNA was not sheared or size-selected for ONT library preparation.

The genome was assembled, annotated, and analyzed as part of the Bioinformatics Lab (MICROBIO 590B) course at the University of Massachusetts Amherst (7). All software was deployed using default parameters unless otherwise specified. Reads were filtered by FiltLong v0.2.1 (8) to retain ~40X coverage (228,000,000 bp), ≥85% mean quality score, and 1,000 base pairs minimum read length. Average read length was determined to be 15,810 bp using SeqFu (9). Reads were assembled using the *de novo* Flye assembler v2.9.2 (10) producing 216,153 reads, then aligned and mapped with Minimap2 v2.26 (11). The draft assembly was corrected with Racon v1.4.3 (12) and polished with Medaka v1.8.1 (13) to generate a final consensus sequence assembly. The final assembly was uploaded into KBase (14), QUAST v4.4 (15), and CheckM v1.0.18 (16) determined the completeness and the contamination (17) (Table 1).

**TABLE 1:** Genome assembly quality assessment values shown, data obtained through QUAST and CheckM^*a*^

Features	RC340
Total base pairs in the assembly	229,161,969
Assembled genome size (bp)	5,868,817
Fold-coverage	39.05
Number of contigs	3
Largest contig (bp)	5,469,113
Assembly N50 (bp)	5,469,113
Assembly L50	1
G + C content (%)	34.99%
Completion (%)	95.91%
Contamination (%)	0.23%

^*a*^ Quality assessment metrics are largest contig, completion, and contamination.

**Fig 1:**
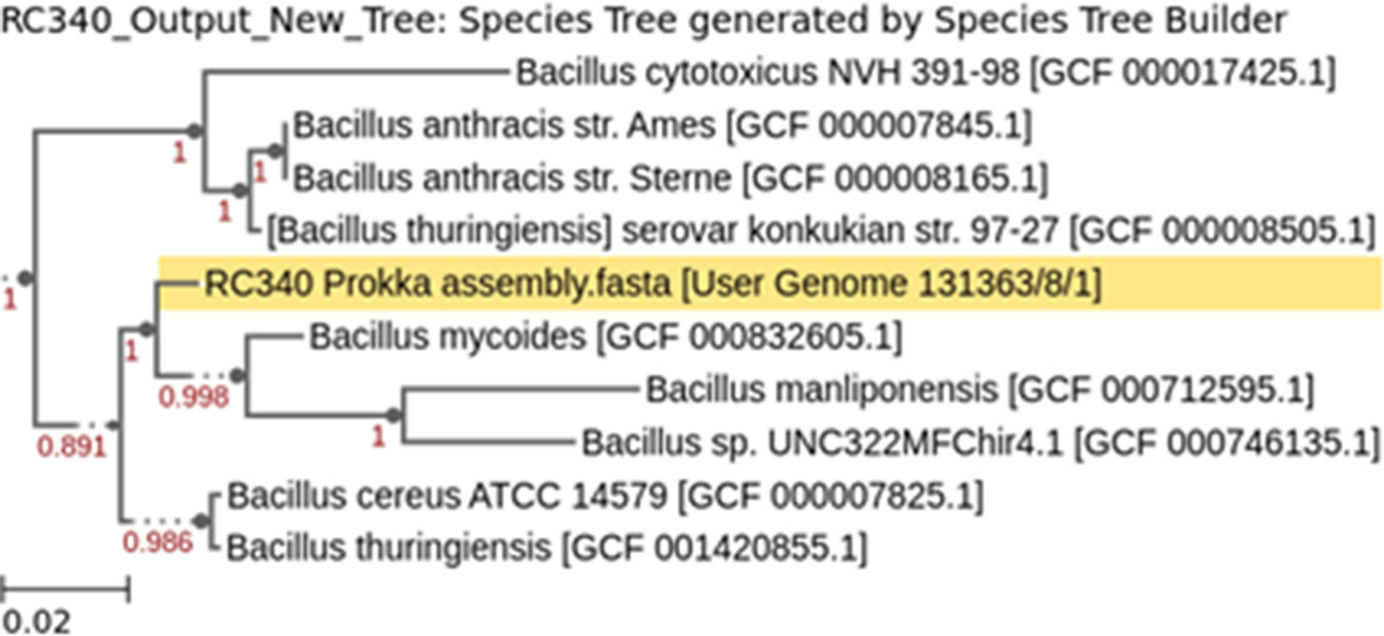
Insert Genome Into SpeciesTree v2.2.0 program created tree with Prokka file as input and a neighbor public genome count of 9, closest phylogenetic neighbors shown above. *B. mycoides*, *B. cereus*, and *B. thuringiensis* are closest neighbors.

The final RC340 assembly file was annotated in KBase using Prokka v1.14.5 (18). A phylogenetic tree was constructed by Insert Genome Into SpeciesTree v2.2.0 (19) with a neighbor public genome count of 9 (Fig. 1).

The taxonomy for RC340 from Classify Microbes with GTDB-Tk v1.7.0 (20) is Domain Bacteria, Phylum Bacillota, Class Bacilli, Order Bacillales, Family Bacillaceae, Genus *Bacillus*, Species *thuringiensis*. Compute ANI with FastANI v0.1.3 (21) was utilized to quantify the average nucleotide identity between RC340 and its nearest neighbor *B. thuringiensis* (GCF 00140855.1). The ANI estimate was 95.865% which classifies RC340 genome as the same species.

To assess the presence of bacitracin resistance genes in the RC340 genome and the nine closest related neighbors, we used Compare Genomes from Pangenomes v0.0.7 (22). The pangenome output contained five bacitracin resistance genes only present in the RC340 genome. These were bacitracin export, resistance, and transport proteins. The *Bt* strain RC340 bacitracin resistance genes may provide a competitive advantage in the battle for nutrients in nutrient-depleted soils due to climate change stress (23, 24).

## Data Availability

The 16S rRNA GenBank sequence accession number is OQ534872. The Sequence Read Archive (SRA) accession number is SRX19731073, and the genome assembly accession number is JARXOY000000000.
